# Prevalence and Determinants of Mobile Health Applications Usage: A National Descriptive Study

**DOI:** 10.3389/fpubh.2022.838509

**Published:** 2022-04-27

**Authors:** Samar A. Amer, Ayah Bahumayim, Jaffer Shah, Nouf Aleisa, Basma M. Hani, Doaa I. Omar

**Affiliations:** ^1^Department of Public Health and Community Medicine, Faculty of Medicine Zagazig University, Zagazig, Egypt; ^2^Member at Royal College of General Practitioners, London, United Kingdom; ^3^Department of Mental Health Primary Care, Faculty of Medicine, Nova University, Lisbon, Portugal; ^4^Health Education Department, College of Applied Medical Sciences, King Saud University, King Khalid University Hospital, Riyadh, Saudi Arabia; ^5^Medical Research Center, Kateb University, Kabul, Afghanistan; ^6^Health Education Department, College of Applied Medical Sciences, The General Directorate of Clinical Health Education Department, Ministry of Health, Riyadh, Saudi Arabia; ^7^Department of Community, Environmental and Occupational Medicine, Faculty of Medicine, Benha University, Benha, Egypt

**Keywords:** mobile health application, e-health, Saudi Arabia, heath care applications, public health policy (PHP)

## Abstract

We aimed to determine the prevalence of MHAs' usage and explore the context and determinants of using MHAs among inhabitants in Saudi Arabia (SA). This cross-sectional study randomly selected 679 adult inhabitants from the 20 health regions in SA through an electronic, self-administered, well-structured, and validated Arabic questionnaire. The prevalence of using MHAs was 47.9%, and it was significantly higher among younger, Saudis, highly educated, and working participants, as well as those with chronic diseases (*p* < 0.05). The main motives for using MHAs were to promote health status (68.6%) and to lose weight (33.2%). The most used apps were related to daily steps-counting (54.2%), and among females was tracking ovulation period apps (43.5%). The most common reported advantage of using MHAs was saving time (64%). Despite the potential benefits of MHAs, they were used by only about half of the study participants in SA. The most effective MHAs in improving health status were exercise, calorie-related, water uptake, and daily steps-counting apps. Policymakers looking to address reform aimed at improving health with mobile apps will find our study interesting.

## Introduction

In the past years, technological advances, including web-based monitoring systems and mobile phone applications (apps), have changed the consumers' ways of accessing healthcare and enabled the use of digital mechanisms for health monitoring and self-care (1). In terms of legal and public policy considerations, E-health (electronic health) has become important (2, 3).

Mobile health (m-health) interventions, such as mobile health apps (MHAs), have become a fast-growing, assistive technological (AT) form of help in wellness monitoring, the prevention and/or management of diseases, and the improvement of health outcomes, as well as with the therapeutic management of chronic illnesses through the involvement of healthcare and active self-management (4, 5). As mentioned in the digital marketing strategy (2012–2020) by the European Commission, e-health plays a crucial role. It states, “When e-health is applied effectively, it delivers more ‘person-centered' healthcare, which is more targeted, effective, and efficient and helps reduce errors, as well as hospitalization length. It facilitates socio-economic inclusion and equality, quality of life and patient empowerment through greater transparency, access to services and information, and the use of social media for health” (2, 3).

Saudi Arabia (SA) is estimated to have 19.4 million smartphone users (6). Mobile (apps) on the two leading platforms, iOS and Android, have become prevalent (7). Medical apps are defined as any software app that is created for medical or other health-related purposes and is used on a mobile device. There are 32,104 healthcare apps available (8), and their use by patients and clinicians has grown dramatically.

In 2005, the World Health Organization (WHO) adopted decision number 28, urging the ratification of e-health as a way to strengthen health systems. The E-Health Initiative is one of forty initiatives under the Saudi National Transition 2020 program. Its goal is to improve the efficiency and effectiveness of healthcare through the adoption of information technology and digital transformation in order to provide SA citizens with standardized digital medical records by 2020 (9). The Sehha app developed by the Saudi Ministry of Health (SMOH) was introduced in 2018 to offer a fast and efficient means of direct consultation with medical experts, regardless of time or place (10).

MHAs potentially promote the self-management of many diseases in daily life. However, adoption of these apps is still limited, and the knowledge about MHAs' efficient utilization in regards to facilitators and barriers is small. Researchers can use mobile apps to discover new enabling, engaging, and empowering methods to help patients interact, access, record events, and engage in the provided care (11).

Therefore, our study is aimed at optimizing the use of MHAs in delivering effective healthcare services through the following objectives among adult inhabitants of Saudi Arabia during February 2020. The goal is to determine the prevalence of MHAs' usage, explore the context of using MHAs (numbers, types, benefits, advantages, and barriers), and study the determinants of MHAs (the relationship between the use of MHAs and demographic characteristics).

## Methodology

### Participants and Study Design

During (February 2020), This cross sectional study targeted inhabitants from the 20 health regions in South Africa who agreed to participate if they met the following selection criteria: Internet users (12); males and females aged from 16 to 60 years (13).

### Sample Size

The sample size was calculated using the Epi-info program (EPI) info website. There was a total of 13,629,686, and after excluding the illiterate population at around 3 million, the target population was 12,948,201. The percentage of Internet users was 91 percent (13), with a similar percentage of smartphone users (14). As a result of our study's power (80%), precision (0.5%), and 95% confidence interval, the calculated sample size from the total population of 11,782,864 was 680 participants after we multiplied it to increase the power of our study by adding more representatives to the studied subjects.

### Data Collection Method

Using a multi-stage sampling method, a community-based sample was used to represent the 13 administrative health regions in SA, weighted per proportion to the population density in each region, with a minimum of thirty participants (4.5%) from each region. Taking into account the 3:1 ratio between the inside and outside of the city. The sample was Riyadh (123), Macca (106), Eastern region (85), Al-Madinah Al Munawara (54), Asser (45), Tabuk (37), Jizan (36), northern border (35), Al-Jouf (33), Al-Qassim (33), Hail (33), Najran (31), and Al-Baha (30) (15). The data was collected through a self-administered, online-designed questionnaire. After signing a written informed consent, the participants completed and submitted the questionnaire.

#### Data Collection Instrument

It was a pre-tested, well-structured, electronic, self-administrated questionnaire. A Google form of the Arabic questionnaire was designed. An assessment schedule was developed by six experts for validating the questionnaire, after which it was tested for clarity and comprehension through a pilot study, which was conducted on 45 participants. Their results were not included in our study results. The calculated Cronbach's alpha was 0.74.

The questionnaire was composed of three main parts, each with a different function, as follows: (1) describing the demographic characteristics of the studied population; (2) collecting data about the prevalence of mobile apps (use, frequency, and types); (3) subjective assessment about the effectiveness (benefits and limitations) of the apps' use.

#### Statistical Analysis

The collected data was coded and analyzed with SPSS (version 27) (16) at a predetermined level of significance (*p* < 0.05). The mean, standard deviation (SD), and range were all used for the summarization of quantitative data such as age, and a *t*-test was used for the analysis. Qualitative data such as sex, level of education, and marital status were presented as frequencies (F) and percentages and analyzed using the Chi-square test. Binary logistic regression was used to predict the role of the following variables: age, level of education, nationality, and co-morbidities, in using MHAs.

#### Ethical Considerations

Participants were provided with written informed consent forms before answering the questionnaire. The questionnaire did not contain any sensitive or private questions, and the participants' identities remained anonymous. The institutional review board of King Fahad Medical City approved this study (IRB Log Number19-244E).

## Results

### Socio-Demographic Data

Our study was carried out on 679 participants, among whom 503 (74.1%) were females, 643 (94.7%) were Saudis, 494 (72.8%) were University-educated, 247 (36.4%) were married, and 290 (42.7%) were working participants, while 295 (43.4%) of participants had comorbidities (Table 1).

**Table 1:** The socio-demographic characteristics of the studied participants, and its relation to the use of mobile health applications (MHAs).

**Demographic variables**	**Total**	**Usage of MHAs**		***P***
	**No = 679**	**Non-users**	**Users**	
		**No = 354(52.1)**	**No = 325(47.9)**	
		***F*** **(%)**	***F*** **(%)**	
**Age (y)**				
Mean+_SD	28.9+_9.2	29.7+_9.9	28.0+_8.2	0.02^*^
Range	(16–65)	16–65	16–61	
**Sex**				
Female	503 (74.1)	264 (74.6)	239 (73.5)	0.79
Male	176 (25.9)	90 (25.4)	86 (26.5)	
**Nationality**				
Saudi	643 (94.7)	341 (96.3)	302 (92.9)	0.04^*^
Non-Saudi	36 (5.3)	13 (3.7)	23 (7.1)	
**Level of education**				
Primary	9 (1.3)	7 (2.0)	2 (0.6)	0.04^*^
Secondary, /high University	83 (12.2)	53 (15.0)	30 (9.2)	
Post-graduates	494 (72.8)	249 (70.3)	245 (75.4)	
	93 (13.7)	45 (12.7)	48 (14.8)	
**Marital status**				
Widow	5 (0.7)	2 (0.6)	3 (0.9)	0.56
Single	404 (59.5)	206 (58.2)	198 (60.9)	
Married	247 (36.4)	136 (38.4)	111 (34.2)	
Divorced	23 (3.4)	10 (2.8)	13 (40.0)	
**Occupation**				
Student	257 (37.8)	127 (35.9)	130 (40.0)	0.003^*^
Employee	290 (42.7)	141 (39.8)	149 (45.8)	
Unemployment	132 (19.4)	86 (24.3)	46 (14.2)	
Co-morbidities	295 (43.4)	149 (42.1)	147 (45.2)	0.03^*^
Had children	223 (34.3)	128 (36.2)	105 (32.3)	0.29

* *p < 0.05. There was a statistical significant difference*.

### Prevalence and Predictors of the Use of MHAs

The prevalence of using MHAs among the studied group was 325 (47.9%). The usage of MHAs was significantly higher among both younger and Saudi participants, as well as among participants who attended the University with a higher level of education, and additionally working participants and participants with chronic diseases. This was in comparison to other groups (*p* < 0.05) (Table 1).

Higher, non-significant odds were observed among those aged 20–30 years, Saudis, those who had received University and post-graduate education, and those who had chronic diseases (OR =1.02, 0.48, 3.01, 3.37, and 1.29 respectively) (Table 2).

**Table 2:** Binary logistic regression of the predictors of MHAs use.

**Variables**	**B**	**S.E**.	**Wald**	**Sig**.	**Exp(B)**	**95% C.I for EXP(B)**
**Age** <20 y(ref)	–	–	–	–	–	-
20– <30 y	0.021	0.276	0.006	0.940	1.021	(0.594–1.755)
30– <40y	−0.188	0.316	0.354	0.552	0.829	(0.446–1.539)
40– <50 y	−0.888	0.427	4.317	0.038	0.412	(0.178–0.951)
50 or more y	−0.527	0.477	1.219	0.269	0.590	(0.232–1.505)
**Nationality** Non–Saudi (ref)	–	–	–	–	–	-
Saudi	−0.733	0.365	4.031	0.045^*^	0.480	(0.235–0.983)
**Level of education** Primary(ref)	–	–	–	–	–	-
Secondary/high	0.645	0.846	0.580	0.446	1.905	(0.363–10.008)
University	1.103	0.826	1.785	0.181	3.014	(0.597–15.205)
Post-graduates	1.215	0.844	2.073	0.150	3.372	(0.645–17.636)
**Co-morbidities With Co-morbidities** (ref)	–	–	–	–	–	-
**Without Co-morbidities**	0.237	0.163	2.123	0.145	1.268	(0.921–1.744)

* *p < 0.05. There was a statistical significant difference*.

### Context and Types of MHAs Used

The majority of MHA users (154, 47.4%) had more than three MHAs on their mobile devices, 243 (74.8%) downloaded these apps themselves, 34.5% used these apps infrequently (3 times per month), and 52.3% reported that using MHAs affected their health status and lifestyle in some way (Table 3).

**Table 3:** Prevalence and context of the usage of MHAs.

**The users of MHAs {T = 325(47.9%)}**	***F*** **(%)**
**The Number of MHAs on your smartphone**	
One MHA	73 (22.2)
2–3 MHAs	98 (29.8)
>3 MHAs	154 (47.4)
**MHAs downloaded by**	
Default in smartphone	30 (9.2)
Myself	243 (74.8)
Both	52 (16.0)
**Frequency of using MHAs**	
Never	22 (6.8)
Rare (<3 times per month)	122 (34.5)
Sometimes (1–2 times/week)	95 (29.2)
Often (3–4 times/week)	58 (17.8)
Usually (5 times or more/week)	38 (11.7)
**Participant sources of information about MHAs**	
Specialist (doctor, specialist, sports coach)	32 (9.8)
Social media	195 (60.0)
Advertisements in other Apps	65 (20.0)
Famous social media influencer	24 (7.4)
Friends or family members	129 (39.4)
Self-Web site search	47 (14.5)
Others (work, and/ or poster on Primary Health Care Centers)	22 (6.8)
**Using MHAs affect your health status and life style**	
No (Disagree)	46 (14.2)
To some extent (Slightly agree)	170 (52.3)
Yes (Agree)	94 (28.9)
Marked/noticeable effect (Strongly agree)	15 (4.6)
**Motivations for using MHAs were to#**	
Practice physical exercise	81 (24.9)
Medical consultation	80 (24.6)
Follow up the water uptake	72 (22.1)
Promote the health status	223 (68.6)
Weight loss	108 (33.2)
Curiosity	39 (12.0)
Others	

*#Multiple answers were allowed*.

The main sources of information about MHAs among the studied participants were social media, friends and relatives, advertising on another app, and self-search [195 (60%), 129 (39.4%), 65 (20.0%), and 47(14.5%), respectively]. The motivations for using MHAs among participants were to promote the health status (68.6%) and lose weight (33.2%) (Table 3).

**Figure 1:**
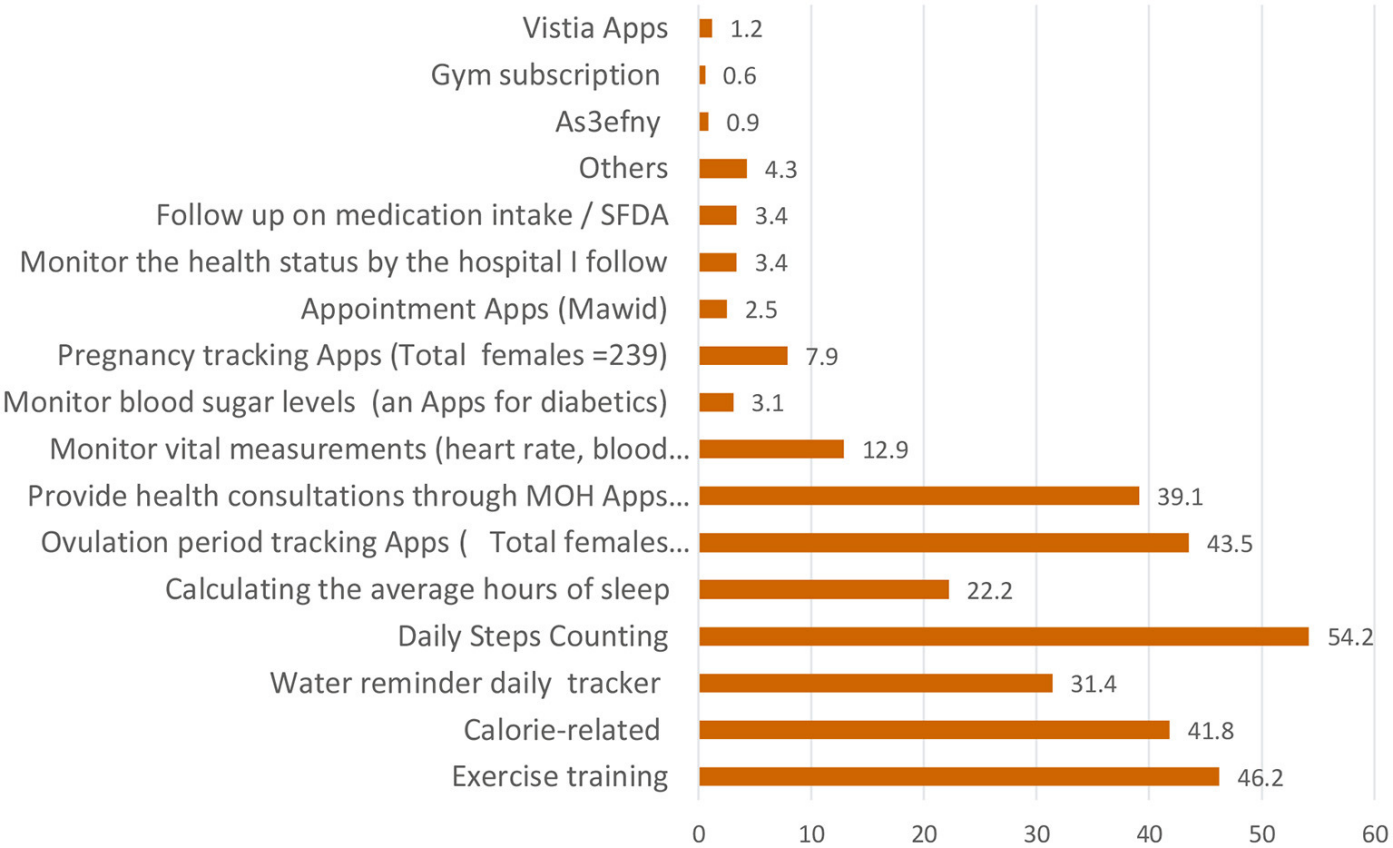
The used mobile health applications (MHAs) among the studied participants.

Our study revealed that a total of 325/679 (47.9%) studied participants used MHAs, with the most commonly used apps being the ones related to daily steps-counting (54.2%). The most commonly used apps by women were the ovulation period tracking apps (43.5%). The least commonly used apps were the Bek Nahtam (0.3%), La Baas (0.3%), and Cura apps (0.3%). The other apps were related to children's health and developmental follow-up (0.3%) (Figure 1).

### Perceived Effectiveness of Using MHAs Among the Studied Participants

The following apps were reported as effective applications in descending order: training exercise, calorie-related, water uptake, and calculating the number of steps per day (48, 45.2, 39.2, and 33.5%, respectively). More than one-third of participants (37.2%) reported that providing health consultations through Ministry of Health (MOH) apps, e.g., Sehha, worked very effectively. Most of the studied participants did not know whether the following apps (calculating the ovulation period, monitoring blood sugar levels, and monitoring vital measurements) were effective (39.1, 38.5, and 37.2%, respectively) (Table 4).

**Table 4:** Public's perceptions about the effectiveness of the most commonly used MHAs among the studied participants.

**Application(apps) related to**	**Harmful *F* (%)**	**Useless *F* (%)**	**Effective *F* (%)**	**Very effective *F* (%)**	**I don't know *F* (%)**
Exercise training	1 (0.3)	13 (4.0)	147 (45.2)	119 (36.6)	45 (13.8)
Calorie-related apps	0 (0.0)	24 (7.4)	156 (48.0)	58 (17.8)	87 (26.8)
Follow up the uptake of water	1 (0.3)	40 (12.3)	129 (39.7)	93 (28.6)	62 (19.1)
Daily steps counting	0 (0.0)	7 (2.2)	108 (33.2)	176 (54.2)	34 (10.5)
Calculating the average hours of sleep	1 (0.3)	46 (14.2)	109 (33.5)	69 (21.2)	100 (30.8)
Ovulation period tracking apps	0 (0.0)	24 (7.4)	81 (24.9)	93 (29.6)	127 (39.1)
Providing health consultations by MOH apps e.g., Sehha	2 (0.6)	13 (4.0)	89 (27.4)	121 (37.2)	100 (30.8)
Monitor vital measurements (heart rate, blood pressure, oxygen level)	5 (1.5)	28 (8.6)	83 (25.5)	84 (25.8)	125 (38.5)
Monitor blood sugar level (apps for diabetics)	4 (1.2)	15 (4.6)	66 (20.3)	93 (28.6)	147 (45.2)

### Advantages and Disadvantages of Using MHAs Among the Studied Participants

The main advantages of using MHAs were saving time (64%), the possibility of following up on the health status at any time (48.9%), and getting correct information (40.9%). The main reported disadvantages included not studying the medical situation thoroughly (63.1%), a lack of continuous follow-up from a specialist (52.9%), and a low quality of diagnosis and follow-up (40.6%). Our study reported that the public's suggestions on how to improve the usage of MHAs included sharing health files or laboratory results with the follow-up specialists (73.5%), linking the health information of the users to their health files (63.3%), following up on health status using charts (56.3%), and adding health information about specific diseases (54.1%) (Table 5).

**Table 5:** Advantages, disadvantages, and public's suggestions of using MHA.

**Among the users of MHAs total = 325(47.9%)**	***F*** **(%)**
**Disadvantages of using MHAs**	
Low quality of diagnosis, and follow-up	132 (40.6)
Do not study the medical situation thoroughly	205 (63.1)
Lack of continuous follow-up from a specialist	172 (52.9)
Expensive	31 (9.6)
App's size is large and therefore takes up space from the mobile	47 (14.5)
I don't know	2 (0.6)
Others	67 (20.6)
**Advantages of using MHAs**	
Saving time and effort.	208 (64.0)
Communicating with the specialist through the App is better than face-to-face communication	66 (20.3)
Possibility to communicate with a specialist through the App at any time	120 (36.9)
Possibility to follow up on the health status at any time	159 (48.9)
Gathering health information.	1 (0.3)
Getting correct information	133 (40.9)
**Participants' suggestions to improve the usage of MHAs**	206 (63.3)
Linking the health information of the user to their health file	176 (54.1)
Adding detailed health information about the diseases of concern	239 (73.5)
Sharing the health file or Laboratory results with the follow-up doctors	183 (56.3)
Chart to follow-up the health status	2 (0.6)
Advertisements to increase the public's awareness about the importance, and uses of different apps Others	33 (10.2)

### Participants' Opinions Regarding the Notifications of MHAs

**Figure 2:**
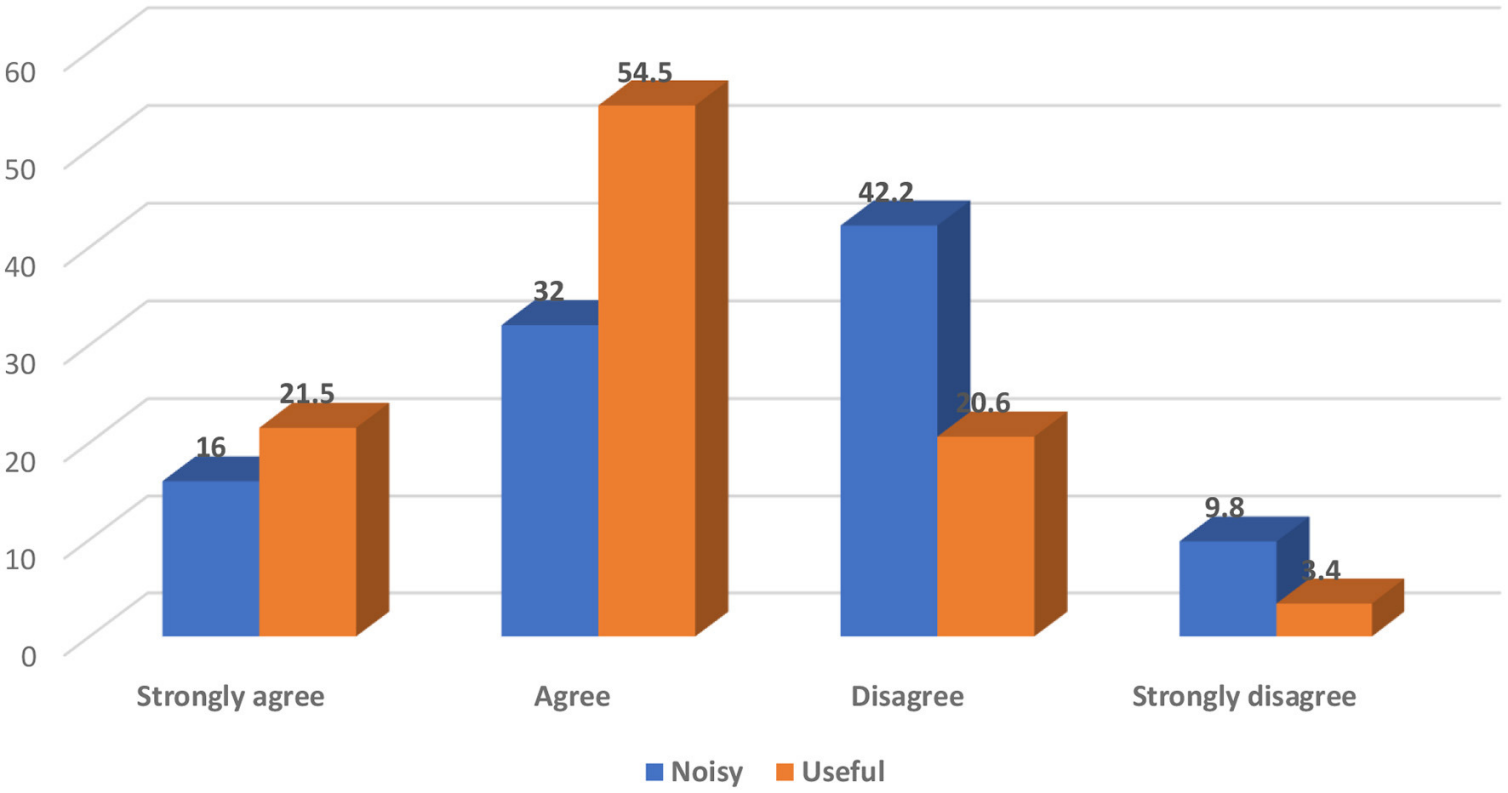
Participants' opinion toward MHAs notifications.

More than half of the studied participants (54.5%) perceived the MHAs' notifications as useful (Figure 2).

## Discussion

This study was conducted to determine the prevalence, context, and determinants of MHAs' usage among 679 participants in SA, who fulfilled the selection criteria. The majority of the participants (74.1%) were females, Saudis (94.7%), had received University or higher education (72.8%), were married (36.4%), employed (42.7%), and had no comorbidity (56.7%).

The current study reported that the prevalence of MHAs' usage was 325 (47.9%) among the studied participants, which was higher than that reported by Bhuvan et al. in a survey that found that the penetration of MHAs was 351 (34.6%) among 1,014 Egyptian patients (17).

The usage of MHAs was significantly higher among participants who were younger, Saudis, had received University education or a higher level of education, were working, or had a chronic disease (*p* < 0.05). Higher, non-significant odds were among those aged 20 to <30 years, Saudis, had received University and postgraduate education, and had chronic diseases (OR = 1.021, 0.480, 3.014, 3.372, and 1.268, respectively). Bol.et al. observed almost comparable results, showing that most MHA users were either younger (OR = 0.97; % CI: 0.96–0.98) or highly educated (OR = 1.12; % CI: 1.01–1.24) than non-users among Dutch populations (18). Nunes et al. discovered that age is a significant predictor of acceptance of information and communication technologies (19). This could be explained by the high e-health literacy level among younger, more educated adults who are technology-savvy.

There was no statistically significant relationship between MHA users and non-users regarding sex, as similar results were reported among a large sample of the Dutch population (OR = 1.25; 95% CI: 0.94–1.66) (18). In contrast to the current results, Xie et al. revealed that females were significantly more likely to use health apps than men, particularly with health and medical reminder apps (20). This is because there are gender differences in attitudes toward healthy living. For instance, women care more about healthy living, healthy eating, and being better adherents to public health recommendations for exercise, tobacco and alcohol consumption, and healthy diets when compared to men. In addition, motherhood might be one reason for using more health apps related to pregnancy, postnatal recovery, and baby care, when compared to men.

In our study, 325 MHA users used more than 21 MHAs. These results are comparable with the results of the Bhuvan et al. study, which reported that 48 MHAs were used by 278 participants, all of whom used different types of MHAs for various purposes, such as multi-purpose health apps, fitness apps, period-tracking apps, meditation apps, and health-monitoring apps (17).

Out of the 325 (47.9%) MHA users studied, 47.4% of them had more than three MHAs on their mobile devices and 74.8% downloaded these apps by themselves. This may have been attributed to the fact that different types of MHAs are available for free *via* the stores of Apple and Google Play (7). This is in addition to the rate of smartphone use being high (6). Also, there are the free Wi-Fi initiatives that allow free Internet roaming for 2 h daily in specific locations, with more than 60,000 free Wi-Fi hotspots in public places across SA, for example, shopping malls, city parks, and hospitals. SA is the region's largest information and communication technologies (ICTs) market, and Riyadh was named the first Arab Digital Capital in 2020 (21).

These findings showed a relatively higher rate of MHA usage than what was reported by Qan'ir et al. who reported that 41.6% of their study participants downloaded at least one MHA, and a maximum being five. Approximately 30% of MHA users (32.4%) reported that they used MHAs a few times per month (22).

The main public sources of information about MHAs were social media 195 (60%), followed by friends and family members (39.4%). This is in agreement with the BBC report about the phenomenon of social media growth in SA reaching the highest social media penetration and making up the largest social media markets in the world. These factors negotiate the use of social media platforms as a proper means of communication and interaction in different contexts (23). Friends and family members recommend the use of MHAs as an essential factor in increasing the apps' credibility and describing the importance of social influence. Therefore, designing health apps to promote wide usage should include adding features publicly demonstrating their usage to a user's close social network. (24). Nearly similar results in another study stated that participants most frequently learned about the MHAs *via* the following sources in descending order: apps stores, friends/family, web searches, and health professionals (74, 25.2, 19.8, and 19.8%, respectively) (22).

The most commonly used apps were related to daily steps-counting (54.2%), training exercise (46.2%), calories (41.8%), and health consultations (39.1%). The popularity of using these apps could be due to the high prevalence of obesity, physical inactivity, and non-communicable diseases in SA (25, 26), which is increasing the public's interest in managing their diets and lifestyles to stay healthy. These factors relate to the easy usability of theses apps, which could increase users' self-efficacy and willingness to use them. In addition to the reported effectiveness of these apps by more than half of MHA users, this comes in accordance with the study by Bhuvan et al. who stated that multi-purpose apps were used as apps for health, fitness, period-tracking, meditation, and health-monitoring (16).

The main motives of using MHAs among the studied MHA users were, in descending order, to promote their health status (223, 68.6%), lose weight (108, 33.2%), and practice physical exercise (81, 24.9%). Because the majority of MHA users said that using the following applications (those for daily step counting, exercising, calorie counting, and health consultations) was helpful and highly effective in improving their health. Therefore, the required features and specifications of MHAs are considered important aspects in expanding the use of MHAs to satisfy the patients' needs and preferences.

The majority of MHA users reported effective use of the majority of commonly used MHAs in improving their health status. These results were even higher than the results reported by a study conducted in China, in which 37.4% of their participants rated their agreement with the statement “using MHAs is effective and can increase knowledge in addition to improving the effectiveness of the management of health conditions” (20). MHAs are considered respected channels of communication in developing countries, serving to effectively complement conventional, efficient communication strategies and improve patients' relationships between individuals and healthcare providers (HCPs) or systems. This is because patients can use the MHAs for various tasks such as requesting medical consultations and information about their health conditions, making appointments, and viewing their medical records (27).

More than half of the MHA users (54.5%) perceived the MHAs' notifications as useful. Notifications are triggered by the apps as reminders and alerts to encourage the efficient use, interact with the participants, and increase the likelihood of achieving the participants' goals. Also, Mendiola et al. reported that mobile apps that are easy to use, simple, have applicable instructions to manage a condition, and help to share data with designated individuals are all deemed to motivate people to be users (28).

Our current study found that the main advantages of using MHAs were saving time (64%), possibility of following up on the health status at any time (48.9%), and getting correct information (40.9%). Nearly similar results in a Malaysian study stated that the main benefits of MHAs were tracking health status (47%), motivation (41%), and gaining knowledge about health and fitness (9%) (11). Mansour in an Egyptian study reported that some of the advantages of using MHAs include apps being reliable (98.9%), free (98.6%), credible (96.3%), friendly to users (94.6%), simple (93%), convenient (92.9%), accurate (92.6%), and secure (90.6%), as well as being able to increase the speed of finding information (97.2%), provide more information (94.3%), build confidence (93.7%), substitute for a doctor (93.4%), help communication (92.6%), and reduce paper use (92.3%) (5).

In our study, the main reported disadvantages of using MHAs were that they, in order, do not study the medical situation thoroughly (63.1%), lack a continuous follow-up from a specialist (52.9%), and have a low quality of diagnosis and follow-up (40.6%), while in China the main reported disadvantages were the apps' inaccuracy (24%), inconvenience (20.7%), and unfriendly use (18.5%) (11).

The studied participants' main suggestions on how to improve the usage of MHAs included sharing the health files or laboratory results with the follow-up doctors (73.5%), linking the users' health information to their health files (63.3%), and following up on health status using charts. While in a qualitative study, Peng et al. found that participants recommended social influence and social competition, intangible and tangible rewards, entertainment, and hedonic factors as reasons that might motivate people to continue using health applications (24).

## Strengths

There was a relatively large sample size used, including 13 different nationalities, representing both inside and outside of all the 13 administrative regions in SA, using a validated questionnaire.

## Limitations

It was a descriptive study using only a self-administrated questionnaire.

## Conclusion

Despite the potential benefits of MHAs, they were used by only about half of the studied participants in SA. The use of MHAs was significantly higher among participants who were younger, Saudis, working, or had a chronic disease. The most effective MHAs in improving health status were apps related to exercise, calories, water uptake, daily steps-counting, and health consultations, all by the MOH Apps. Future research to explore the pros and cons of MHAs' usage is recommended.

## Recommendations

To optimize the role of MHAs in delivering effective healthcare services, the integration of many efforts is required, and so we recommend the following: (1) national health education campaigns to increase awareness about the importance, benefits, and types of MHAs, especially among groups who are less likely to use MHAs; (2) using social media and healthcare providers as marketing tools for the newly launched MHAs; (3) developing the MHAs to meet the public's needs and expectations and improve the apps' usability even with people who have little knowledge about mobile technology; (4) encouraging opportunities for researchers, patients, and legislators to work together to improve the usage of MHAs in order to promote and control the health statuses among the public; (5) having health organizations be able to identify high-quality health apps; (6) developing standard evaluation criteria for choosing health apps.

## Data Availability Statement

The raw data supporting the conclusions of this article will be made available by the authors, without undue reservation.

## Ethics Statement

Ethical review and approval was not required for the study on human participants in accordance with the local legislation and institutional requirements. The patients/participants provided their written informed consent to participate in this study.

## Author Contributions

SA: conceptualization. SA, NA, and AB: data curation. SA and JS: analysis. NA and AB: investigation and project administration. SA and DO: methodology and supervision. JS: software. SA and AB: validation. DO, BH, and JS: visualization. SA, DO, BH, and JS: writing—original draft preparation. All authors: writing—review and editing. All authors contributed to the article and approved the submitted version.

## Conflict of Interest

The authors declare that the research was conducted in the absence of any commercial or financial relationships that could be construed as a potential conflict of interest.

## Publisher's Note

All claims expressed in this article are solely those of the authors and do not necessarily represent those of their affiliated organizations, or those of the publisher, the editors and the reviewers. Any product that may be evaluated in this article, or claim that may be made by its manufacturer, is not guaranteed or endorsed by the publisher.

## Abbreviations

apps: Mobile applications
MHAs: Mobile Health Applications
m-health: Mobile health
AT: Assistive technology
e-health: Electronic health
SFDA: Saudi Food and Drug Administration
SMOF: Saudi Ministry of Health
SD: Standard deviation
HCPs: Healthcare providers
King Fahad Medical City
MOH: Ministry of health
ICTs: Information and communication technologies
C.I: Confidence Interval
F: Frequency
HCPs: Healthcare providers
OR: Odds Ratio
Fig: Figure.
